# Proteomic Analyses of Autologous Chondrocyte Implantation Plasma Highlight Cartilage Acidic Protein 1 as a Candidate for Preclinical Screening

**DOI:** 10.1177/03635465231156616

**Published:** 2023-04-11

**Authors:** Charlotte H. Hulme, Mandy J. Peffers, Sally Roberts, Pete Gallacher, Paul Jermin, Karina T. Wright

**Affiliations:** * Centre for Regenerative Medicine Research, School of Pharmacy and Bioengineering, Keele University, Keele, UK; † Robert Jones and Agnes Hunt Orthopaedic Hospital Foundation Trust, Oswestry, UK; ‡ Institute of Life Course and Medical Sciences, University of Liverpool, Liverpool, UK; Investigation performed at Keele University, Keele, UK, and Robert Jones and Agnes Hunt Orthopaedic Hospital, Oswestry, UK

**Keywords:** plasma, biomarkers, predictive, autologous chondrocyte implantation, proteomics

## Abstract

**Background:**

Stratification is required to ensure that only patients likely to benefit receive autologous chondrocyte implantation (ACI). It would be advantageous to identify biomarkers to predict ACI outcome that are measurable in blood, avoiding the need for an invasive synovial fluid harvest.

**Purpose:**

To assess if proteomic analyses can be used to identify novel candidate blood biomarkers in individuals who respond well or poorly to ACI.

**Study Design:**

Controlled laboratory study.

**Methods:**

Isobaric tagging for relative and absolute quantitation (iTRAQ) mass spectrometry was used to assess the proteome in plasma pooled from ACI responders (mean Lysholm improvement after ACI, 33; n = 10) or nonresponders (mean, −13; n = 10), collected at the time of surgery for cartilage harvest (stage 1) or implantation of culture-expanded chondrocytes (stage 2). An alternative proteomic method, label-free quantitation liquid chromatography–tandem mass spectrometry, was used to analyze plasma samples (majority matched to iTRAQ) individually. Differentially abundant proteins (±2.0-fold) were analyzed from both proteomic data sets, and markers of interest identified via pooled iTRAQ were validated via immunoassay of individual samples.

**Results:**

Protein differences could be detected in the plasma preoperatively between ACI responders and nonresponders (16 proteins; ≥±2.0-fold change; *P* < .05) using iTRAQ proteomics. The most pronounced plasma proteome shift was evident in response to stage 1 surgery in ACI nonresponders, with 48 proteins being differentially abundant between the procedures. Label-free quantitation liquid chromatography–tandem mass spectrometry analysis of these same plasma samples (nonpooled) resulted in very few proteins being identified that were significantly differentially abundant. However, this work highlighted cartilage acidic protein 1 as being increased preoperatively in nonresponders as compared with responders.

**Conclusions:**

This study is the first to use proteomic techniques to profile the plasma of individuals treated with ACI. Despite iTRAQ analysis of pooled plasmas indicating that there are differences in the plasma proteome between responders and nonresponders to ACI, these findings were not replicated when assessed using an alternative nonpooled technique. This study highlights some of the difficulties in profiling the plasma proteome in an attempt to identify novel biomarkers. Regardless, cartilage acidic protein 1 has been identified as a protein candidate, which is detectable in plasma and can predict outcome to ACI before treatment.

**Clinical Relevance:**

Candidate plasma protein biomarkers identified in this study have the potential to help determine which patients will be best suited to treatment with ACI.

Autologous chondrocyte implantation (ACI) is a cell therapy approach used to treat chondral/osteochondral defects.^10^ ACI requires two surgical procedures: in the first (stage 1), cartilage is harvested from a low weightbearing region of the joint, from which chondrocytes are extracted via enzymatic digestion and then culture expanded for 3 to 4 weeks before implantation into the chondral/osteochondral defect in a second procedure (stage 2). ACI is cost-effective if used on patients who are likely to benefit.^20,21^ There is, however, a requirement to identify individuals who have a predisposition for failure to prevent them from undergoing two surgical procedures and a significant time of recovery and rehabilitation where ACI is unlikely to yield clinical benefit. Thus, preclinical screening could help to ensure that these patients can be stratified for alternative therapeutic interventions more quickly to prevent the worsening of symptoms and osteoarthritis (OA) progression, in line with the “getting it right first time” scheme.^18^

We previously demonstrated that protein differences exist within the synovial fluid of those who either show clinical improvement after ACI (responders) or not (nonresponders).^11,12,34^ Despite this, there remains a need to identify biomarkers that have the capacity to predict clinical outcome after ACI and other orthopaedic interventions and can be detected within the blood.^14^ Identification of blood biomarkers would have much greater clinical utility, with collection of blood samples for analysis being easier and more widely available, including within the outpatient setting.

This study aimed to assess whether proteome differences exist in the blood between individuals who respond (responders) and who do not (nonresponders) 12 months after ACI. In assessing the plasma proteome, we anticipated that it would be difficult to detect protein differences between response groups that were measurable within the systemic circulation and with the capacity to reflect the damaged joint environment. The identification and translation of plasma clinical biomarkers are widely recognized to represent a challenging undertaking.^6^ In spite of this, work in other fields of orthopaedics has demonstrated that blood proteins can be predictive of clinical outcomes, perhaps by providing a better understanding of an individual's physiology and general health status.^1,7^

Plasma proteins that have differential abundance between responders and nonresponders could be clinically useful candidate biomarker proteins for the prediction of ACI outcome.

## Methods

### Patients

Plasma samples were analyzed from patients identified as responders or nonresponders to ACI. Responders were defined as having a minimum clinically important differenceon the Lysholm score^19,28^ at 12 ± 3 months (mean ± SD) posttreatment, where they had an increase of at least 12 points.^4,26^ The Lysholm score ranges from 0 to 100, where 100 represents perfect knee function. Patients were selected from our biobank cohort to include those who demonstrated the most marked improvements at 12 months as compared with preoperatively (mean improvement, 35 ± 13) and those whose function worsened after treatment (mean change in Lysholm, −13 ± 10 points). We previously showed that selection of these “extremes” of clinical response is helpful for screening proteins that relate to clinical outcome and that can serve as candidates for assessment as prognostic biomarkers in a more heterogeneous population in terms of clinical responses.^11,13^ These studies indicated that 10 samples per group provided sufficient power to detect differentially abundant proteins between groups.^11,13^ Plasma samples were analyzed that were collected immediately before stage 1 and/or stage 2 surgery (Table 1). Specifically, plasma samples were collected by venipuncture at the time of anesthesia for surgery and before any surgical intervention. Note that samples from each time point (ie, stage 1 or 2) were analyzed independently for all analyses. For the isobaric tagging for relative and absolute quantitation (iTRAQ) and label-free analyses, 10 plasma samples were included for each of the 4 biological groups: responders, stages 1 and 2; nonresponders, stages 1 and 2. For the iTRAQ analysis, 7 responders and 6 nonresponders had matched stage 1 and 2 samples analyzed. For the label-free proteomic analysis, patient samples that were matched to those analyzed in the iTRAQ proteomics were prioritized for analysis; however, non–freeze/thawed samples were not available for all patients. Therefore, patient-matched stage 1 and 2 samples were analyzed where possible. Matched stage 1 and 2 samples were analyzed using label-free proteomics for 10 responders and 9 nonresponders. Across the two proteomic techniques, 11 of the same patients had samples analyzed via label-free and iTRAQ; this equated to matched samples for each biological group as follows: 5 nonresponders, stage 1; 7 nonresponders, stage 2; 7 responders, stage 1; 5 responders, stage 2.

**Table 1:** Patient Information^*a*^

					*P* Value	
	Responders		Nonresponders		Responder vs Nonresponder	Stage 1 vs Stage 2
Variable^*b*^	Stage 1 (n = 13)	Stage 2 (n = 15)	Stage 1 (n = 15)	Stage 2 (n = 13)	Stage 1; Stage 2	Responder; Nonresponder
Age, y	38 [6]	37 [11]	39 [13]	38 [12]	.90; .90	.67; .74
Male	12	14	11	9	.32; .32	>.99; >.99
Body mass index, kg/m^2^	30 [6]	30 [7]	27 [6]	27 [6]	.21; .50	.74; .86
Change in Lysholm score	35 (17-58)	35 (17-58)	−14 (−4 to −46)	−14 (−4 to −46)	<.0001; <.0001	.94; .79
Smoker	1	1	0	0	>.99; >.99	>.99; .48
Total defect area, cm^2^	6.25 [4.00]	6.80 [5.78]	5.05 [2.81]	5.52 [3.57]	.56; .56	>.99; .88
No. of defects	1 [1]	1 [1]	2 [1]	2 [1]	.87; .62	.99; .69
Location of defect						
Patella	4	6	8	6	.28; >.99	.71; >.99
Lateral femoral condyle	3	4	1	1	.31; .33	>.99; >.99
Lateral tibial plateau	2	3	0	1	.21; .60	>.99; .46
Medial femoral condyle	6	5	7	7	>.99; >.99	>.99; >.99
Medial tibial plateau	0	0	2	3	.48; .09	>.99; .64
Trochlea	7	7	5	4	.45; .46	>.99; >.99
No. of concomitant procedures	1	6	1	6	>.99; >.99	.03^*c*^; .08
No. of previous operations	1 [0]	1 [2]	1 [2]	1 [1]	.95; .83	.94; .89

a Demographic data for patients treated with autologous chondrocyte implantation who either responded well clinically (responders) or demonstrated poor clinical outcomes (nonresponders) based on change in 12-month postoperative vs preoperative Lysholm score. Plasma was collected immediately before stage 1 or 2 surgery. Demographic differences were assessed between responders and nonresponders at each time point and between time points (stages 1 and 2) in each clinical response group (Mann-Whitney *U* test).

b Data are presented as median [IQR], mean (range), or No.

c *P* < .05.

### Plasma Sample Collection and Storage

Blood samples were collected by venipuncture into blood tubes containing K3EDTA (Vacuette) immediately before surgery at stages 1 and 2 of ACI. Blood samples were then centrifuged at 6000*g* for 15 minutes at 4°C to separate the plasma. The plasma was then divided into aliquots and stored at −196°C in liquid nitrogen before analyses.

### Proteomic Analysis Rationale

To determine whether protein differences could be detected in the plasma between responders and nonresponders to ACI, we carried out a preliminary investigation using iTRAQ proteomic analysis of pooled plasma samples (10 responders, 10 nonresponders). iTRAQ is a method of labeling peptides such that different biological groups are labeled with different weighted tags. This allows for peptide samples from multiple biological groups to be combined and run through the mass spectrometer as a single sample, reducing the instrument running time and hence reducing cost. After mass spectrometry analysis of the combined sample, the different peptides can be computationally reassigned to their affiliated biological group based on the weighted tag, allowing for comparisons to be made in the relative abundance of peptides across biological groups.^33^ This provided an inexpensive, quick analysis that utilized minimal amounts of important clinical samples. Once we demonstrated that plasma proteins could be detected that were differentially abundant between the ACI responders and nonresponders, we attempted to validate a protein difference of interest in the same individual samples using enzyme-linked immunosorbent assays (ELISAs); we also conducted an independent proteomic analysis of nonpooled samples (10 responders, 10 nonresponders) via label-free quantitation liquid chromatography–tandem mass spectrometry (LF LC-MS/MS).

For both proteomic analyses, plasma samples were first protein equalized using ProteoMiner beads in an attempt to overcome the difficulty of low-abundance protein detection in plasma.^15,22^

### Sample Preparation and Analysis Using iTRAQ Proteomics (iTRAQ LC-MS/MS)

Plasma protein concentrations were calculated using a Pierce 660-nm protein assay (Thermo Scientific),^30^ and 5 mg of protein was loaded onto ProteoMiner beads (BioRad) for each sample (n = 40), following kit instructions. Briefly, samples were bound to the beads for 2 hours, washed 3 times to remove any unbound material, and eluted into elution buffer. Twelve microliters was taken from each sample and pooled into each of the experimental groups: stage 1 responders (n = 10), stage 1 nonresponders (n = 10), stage 2 responders (n = 10), and stage 2 nonresponders (n = 10). Pooled samples were stored at −20°C before preparation for iTRAQ analysis.

Pooled protein normalized plasma samples were thawed and then precipitated in 6 volumes of ice-cold acetone for 3 hours at −20°C. Precipitates were pelleted by centrifugation at 13,000*g* for 10 minutes at 4°C and resuspended in 100 μL of triethylammonium bicarbonate buffer. An overall 100 µg of protein for each experimental sample was then subjected to reduction, alkylation, and digestion following instructions on the iTRAQ labeling kit (Applied Biosystems). After overnight trypsin digestion at 37°C, samples were labeled with iTRAQ tags. The following tags were added to each experimental group: 114–, stage 1 nonresponders; 115–, stage 2 responders; 116–, stage 2 nonresponders; 117–, stage 1 responders. The labeled tryptic peptides were then combined, dried down using a vacuum centrifuge, and then stored at −80°C before being analyzed.

iTRAQ-labeled peptides were subjected to liquid chromatography–tandem mass spectrometry (LC-MS/MS) as a service by St Andrew's University Proteomic Facility. Samples were analyzed as described previously.^9,11^ Briefly, samples were added to loading buffer Ascx (10mM monopotassium phosphate [KH_2_PO_4_], 20% acetonitrile [MeCN], pH 3.0) and sonicated, and 0.5M orthophosphoric acid (H_3_PO_4_) was used to adjust the pH to 3.0. Strong cation exchange chromatography was used to separate the peptides, and each fraction was analyzed by nano liquid chromatography–electrospray ionization tandem mass spectrometry using a TripleTOF 5600 tandem mass spectrometer (ABSciex).

ProteinPilot 4.5 software with the Paragon and ProGroup algorithms (ABSciex) against the human sequences in the Swiss-Prot database was used to analyze the raw mass spectrometry data. Searches were performed using the preset iTRAQ settings, with trypsin selected as the cleavage enzyme, with methyl methanethiosulfonate modification of cysteines “bias” and “background” corrections selected, and with a “thorough ID” search effort. ProteinPilot's bias correction assumes that most proteins do not change in expression. Finally, detected proteins were reported with a protein threshold (unused ProtScore [confidence]) >0.05 and used in the quantitative analysis if they were identified with ≥2 peptides with ≥95% confidence. *P* values for the iTRAQ ratios were calculated by the ProteinPilot software. Proteins with iTRAQ ratios with *P* values ≤.05 and with differential abundances ≥±2.0 fold change (FC) were used for further analysis.

### Validation of iTRAQ Proteomic Findings Using ELISA

To verify the mass spectrometry findings, a protein of biological interest was selected and measured by ELISA in the nonpooled ProteoMiner-normalized samples. Our group previously indicated that the acute-phase response signaling pathway is differentially regulated after stage 1 surgery in nonresponders to ACI, as detected in the synovial fluid.^11^ We were interested to determine whether changes in this functional pathway were detectible in the plasma as an indicator of the biological response within the knee joint. As proteins of the acute-phase response signaling pathway demonstrated differential abundance in the plasma of nonresponders at stage 2 versus stage 1 surgery, we therefore selected two of these for validation: clusterin and complement factor H. Furthermore, clusterin was uniquely differentially abundant between stages 1 and 2 of ACI in nonresponders, thereby having greater potential as a marker to differentiate between these clinical outcome cohorts. Clusterin and complement factor H were assessed with a human DuoSet ELISA kit (R&D Systems). Samples were assayed using a 1:10,000 dilution in assay sample diluent for clusterin and complement factor H. The ELISA was carried out according to the manufacturer's instructions. Cartilage oligomeric matrix protein (COMP) has known biological relevance to OA and cartilage injury/repair and has been widely studied as a plasma biomarker of OA progression/structural change^24^; therefore, this was also assessed via immunoassay. Levels of plasma COMP were assessed with an ELISA (BioVendor Laboratory Medicine), as described previously.^34^ Statistical analysis was performed in Prism (Version 9.0; GraphPad). After confirmation of normal distribution using a Kolmogorov-Smirnov test, a Student *t* test was used to assess differential abundance.

### Sample Preparation and Analysis Using Label-Free Quantitation Proteomics (LF LC-MS/MS)

Samples were subjected to dynamic range compression, and on-bead digestion was performed. Briefly, total protein was quantitated as indicated previously, and 5 mg of total protein was added to ProteoMiner beads. Bead-bound proteins were washed and then treated with 0.05% (wt/vol) RapiGest (Waters) in 25mM ammonium bicarbonate for 10 minutes at 80°C before reduction, alkylation, in situ protein digestion without removal of the beads, and acidification of trifluoroacetic acid, as described previously.^12,23^ The peptide-containing supernatant fractions were frozen at −80° before LC-MS/MS.

Peptide-containing fractions were analyzed with a 2-hour gradient on a NanoAcquity ultraperformance LC (Waters) coupled online to a Q-Exactive Quadrupole-Orbitrap instrument (Thermo-Fisher Scientific) as described previously.^12^ ProgenesisQI software (Waters) was used to label-free quantify the raw files of the acquired spectra. As described previously,^12^ the top 5 spectra for each feature were exported from ProgenesisQI and utilized for peptide identification with a locally implemented Mascot server (Version 2.3.01), searching against the Unihuman reviewed database. Search parameters used were as follows: peptide mass tolerances, 10 ppm; fragment mass tolerance, 0.01 Da; ions, 2+ and 3+; missed cleavages, 1; enzyme, trypsin; and instrument type, electrospray ionization–fluorescein isothiocyanate. Modifications were as follows: fixed, carbamidomethyl cysteine; variable, oxidation of methionine, lysine, or proline. The peptide matches above an identity threshold were adjusted to give a false discovery rate of 1% before the protein identifications were reimported into ProgenesisQI for the label-free relative quantification using unique peptides only. Statistical analysis was performed with ProgenesisQI software. Briefly, transformed normalized abundances were used for 1-way analysis of variance, and all peptides (with *P* < .05) of an identified protein were included. Proteins that demonstrated a mean FC ≥±2.0 in abundance between biological comparator groups were reported.

### Ethics Approval and Consent to Participate

Patient samples were collected under the following ethical approvals: “Investigating the potential for cells and molecules isolated from orthopaedic patients for modelling and understanding pathogenic conditions and developing diagnostic markers and therapies for musculoskeletal disorders and spinal cord injury” (11/NW/0875), “Autologous cell therapy for osteoarthritis: An evaluation of the safety and efficacy of autologous transplantation of articular chondrocytes and/or bone marrow derived stromal cells to repair chondral/osteochondral lesions of the knee” (11/WM/0175), and “Arthritis and cartilage repair study” (06/Q6201/9). 11/NW/0875 was approved by the NRES committee, North West–Liverpool East. 11/WM/0175 was approved by the NRES committee, West Midlands–Coventry and Warwick. 06/Q2601/9 was approved by the local research ethics committee, Shropshire and Staffordshire-Shropshire. All patients gave valid informed consent before samples were collected.

## Results

### Patients

Demographic information for the patients is included in Table 1. No demographic parameters were significantly different between responders and nonresponders at stage 1 or 2 or between stage 1 and 2 in responders or nonresponders (*P* > .05; Mann-Whitney *U* test), except the difference in Lysholm score (Table 1). In clinical responders, the number of concomitant therapies at stage 1 was different than at stage 2, reflecting the arthroscopic stage 1 procedure cf. ’open’ stage 2 procedure (Table 1).

### Identification of Proteins With Potential to Predict ACI Outcome Preoperatively

iTRAQ proteomic analysis (pooled samples) identified 16 proteins that were differentially abundant (≥±2.0 FC; *P* < .05) in the plasma between individuals who demonstrated good and poor responses to ACI (Table 2). The proteins with the greatest differential abundance were serotransferrin and Ig gamma 1 chain C region, which were elevated 7.5 and 5.7 FC in responders, respectively.

**Table 2:** Baseline (Stage 1) Differentially Abundant Proteins Between Nonresponders and Responders Identified Using iTRAQ or Label-Free LC-MS/MS^*a*^

Protein With Differential Abundance		iTRAQ Proteomics		Label-free Proteomics	
Description	Accession	Fold Change^*b*^	*P* Value^*c*^	Fold Change^*b*^	*P* Value^*c*^
**iTRAQ proteomics**					
Phosphatidylinositol-glycan-specific phospholipase	P80108	−4.0	**7.56E-06**	1.1	.677
Protein Z-dependent protease inhibitor	Q9UK55	−2.5	**.007**	1.2	.290
Antithrombin III	P01008	−2.4	**.001**	1.1	.974
Apolipoprotein A-V	Q6Q788	−2.3	**.009**	−1.3	.388
Coagulation factor XIII B chain	P05160	−2.0	**.007**	1.5	.233
Complement factor H-related protein 5	Q9BXR6	−2.0	**.012**	−1.6	.935
Fibronectin	P02751	−2.0	**.001**	1.2	.762
C4b-binding protein alpha chain	P04003	−2.0	**.001**	1.0	.806
Fibulin 1	P23142	2.9	**.021**	−1.3	.079
Cartilage oligomeric matrix protein	P49747	3.4	**.001**	−1.2	.450
Complement factor H	P08603	3.5	**8.46E-07**	−1.0	.735
Lysozyme C	P61626	3.6	**.013**	−2.1	.137
Thrombospondin 1	P07996	3.8	**8.53E-05**	−1.1	.783
Platelet basic protein	P02775	5.3	**.037**	1.3	.861
Ig gamma-1 chain C region	P01857	5.7	**.024**	1.0	.733
Serotransferrin	P02787	7.5	**1.75E-08**	−1.2	.526
**Label-free proteomics**					
Cartilage acidic protein 1	Q9NQ79	−1.25	.308	−4.0	**.003**

a Proteins identified by iTRAQ or label-free LC-MS/MS as differentially abundant (≥±2.0-fold change; *P* < .05; protein identified by at least 2 unique peptides) at stage 1 (immediately before surgery) in the plasma of clinical nonresponders vs responders. Proteins identified by iTRAQ LC-MS/MS (pooled samples) were subsequently assessed in the label-free LC-MS/MS data set of matched nonpooled samples. iTRAQ, isobaric tagging for relative and absolute quantitation; LC-MS/MS, liquid chromatography–tandem mass spectrometry.

b Positive numbers denote higher abundance in responders vs nonresponders.

c Bold *P* values denote significant findings.

**Figure 1.**
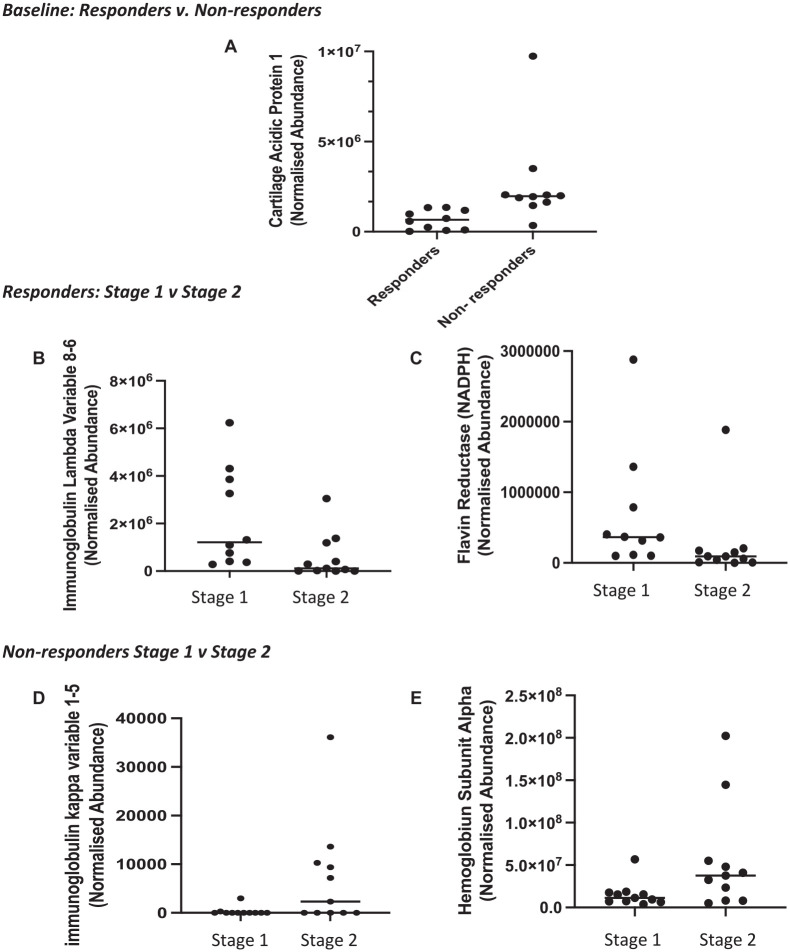
Proteins that were identified as significantly differentially abundant (*P*< 0.05; ≥±2.0-fold change) from the label-free analysis (liquid chromatography–tandem mass spectrometry). (A) Cartilage acidic protein 1 was increased in nonresponders vs responders preoperatively. In ACI responders, (B) immunoglobulin lambda variable 8-6 and (C) flavin reductase (NADPH) decreased immediately before stage 2 surgery (cartilage implantation) as compared with preoperative stage 1. In ACI nonresponders, (D) immunoglobulin kappa variable 1-5 and (E) hemoglobin subunit alpha were increased immediately before stage 2 surgery (cartilage implantation) as compared with preoperative stage 1 surgery. Horizontal line indicates median. ACI, autologous chondrocyte implantation.

Label-free proteomic analysis, however, identified only a single protein, cartilage acidic protein 1, which was significantly increased in nonresponders versus responders preoperatively (−4.0 FC; *P* = .003). Furthermore, this technique identified all the proteins that were identified with iTRAQ analysis, but assessment of individual samples demonstrated that none of these were significantly differentially abundant between responders and nonresponders (Table 2). Figure 1A shows the range of abundances of cartilage acidic protein 1 in the 2 response groups.

### Proteins With Differential Abundance Between Stages 1 and 2 in ACI Responders

In individuals who responded to ACI, iTRAQ proteomics identified 31 proteins that demonstrated a significant differential abundance ≥±2.0 FC between stages 1 and 2 (Table 3). Fourteen proteins were increased in the plasma immediately before stage 2 surgery in comparison with preoperative concentrations. These include the proteins hemoglobin subunits alpha and beta, hemopexin, and serotransferrin (Table 3).

**Table 3:** Responder Differentially Abundant Proteins at the Time of Stage 2 vs Stage 1^*a*^

Protein With Differential Abundance		iTRAQ Proteomics		Label-free Proteomics	
Description	Accession	Fold Change^*b*^	*P* Value^*c*^	Fold Change^*b*^	*P* Value^*c*^
**iTRAQ proteomics**					
Serotransferrin	P02787	−17.2	**.000**	−1.4	.121
Hemoglobin subunit beta	P68871	−14.1	**7.89E-06**	1.7	.241
Hemoglobin subunit alpha	P69905	−13.0	**.003**	2.1	.227
Vitamin D-binding protein	P02774	−7.0	**.001**	−1.3	.705
Thrombospondin 1	P07996	−5.5	**1.66E-08**	1.78	.309
Alpha-1 antitrypsin	P01009	−5.2	**4.54E-06**	1.1	.743
Hemopexin	P02790	−4.9	**.005**	−1.3	.838
Alpha-1 antichymotrypsin	P01011	−4.6	**.004**	1.0	.691
Haptoglobin	P00738	−4.6	**.001**	−1.2	.246
Alpha-2 macroglobulin	P01023	−4.2	**1.46E-09**	−1.3	.785
Thrombospondin 4	P35443	−4.0	**.003**	1.0	.774
Histidine-rich glycoprotein	P04196	−3.2	**.003**	1.1	.599
Coagulation factor XIII B chain	P05160	−3.0	**1.20E-07**	1.9	.224
Complement C3	P01024	−2.8	**1.82E-06**	−1.3	.833
Inter-alpha-trypsin inhibitor heavy chain H1	P19827	2.0	**.016**	−1.1	.834
Cartilage acidic protein 1	Q9NQ79	2.0	**.044**	−1.4	.402
Vitamin K–dependent protein S	P07225	2.1	**.001**	1.1	.788
Complement C1s subcomponent	P09871	2.6	**3.89E-06**	1.2	.466
Beta-Ala-His dipeptidase	Q96KN2	3.1	**.011**	−1.1	.910
Serum paraoxonase/lactonase 3	Q15166	3.1	**.048**	1.1	.567
Alpha-2-HS-glycoprotein	P02765	3.2	**.007**	−1.2	.770
Complement component C9	P02748	3.2	**.001**	−1.5	.700
Phospholipid transfer protein	P55058	3.3	**.013**	−1.0	.870
Complement C1r subcomponent	P00736	3.3	**.001**	1.1	.397
Coagulation factor X	P00742	3.5	**.002**	1.2	.524
Plasma kallikrein	P03952	3.7	**.004**	−1.1	.641
Proteoglycan 4	Q92954	4.3	**.014**	1.2	.682
Inter-alpha trypsin inhibitor heavy chain H2	P19823	4.5	**2.04E-05**	−1.1	.920
Heparin cofactor 2	P05546	4.7	**.0014**	−3.0	.274
Plasminogen	P00747	5.0	**.0001**	1.1	.473
Hyaluronan-binding protein 2	Q14520	7.5	**6.50E-05**	1.1	.844
**Label-free proteomics**					
Immunoglobulin lambda variable 8-6	A0A075B6I0	Not identified	—	3.7	**.027**
Flavin reductase (NADPH)	P30043	Not identified	—	2.8	**.020**

a Proteins that were identified by iTRAQ or label-free LC-MS/MS as differentially abundant (≥±2.0-fold change; *P* < .05; protein identified by at least 2 unique peptides) in the plasma of clinical responders at stage 2 vs stage 1. Proteins identified with iTRAQ LC-MS/MS (pooled samples) were subsequently assessed in the label-free LC-MS/MS data set of matched nonpooled samples. iTRAQ, isobaric tagging for relative and absolute quantitation; LC-MS/MS, liquid chromatography–tandem mass spectrometry.

b Positive numbers denote higher abundance at stage 1 vs stage 2.

c Bold *P* values denote significant findings.

When assessed in nonpooled samples using LF LC-MS/MS, none of these proteins demonstrated different abundance between stages 1 and 2 of ACI. LF LC-MS/MS revealed that only 2 proteins were differentially abundant at 3 to 4 weeks after cartilage harvest surgery versus before. Both these proteins decreased at the time of stage 2 surgery as compared with presurgery (Table 3). Flavin reductase (NADPH) was 2.8-fold higher in the plasma preoperatively versus stage 2 in individuals who responded well to ACI. Furthermore, immunoglobulin lambda variable 8-6 was increased 3.7-fold before any surgical intervention in comparison with the time of stage 2 surgery. Figure 1, B and C, shows the range of abundances of NADPH and immunoglobulin lambda variable 8-6 in each of the ACI stages in responders.

### Proteins With Differential Abundance Between Stages 1 and 2 in ACI Nonresponders

The iTRAQ data indicated that clinical nonresponders of ACI demonstrated the greatest number of proteins with differential abundance immediately before cartilage implantation surgery (stage 2) as compared with baseline (immediately before stage 1 surgery) (Table 4). The LF LC-MS/MS data, however, revealed only 2 proteins that were differentially abundant in the plasma between stages 1 and 2 of ACI in nonresponders. Figure 1, D and E, shows the range of abundances of these—immunoglobulin kappa variable 1-5 and hemoglobin subunit alpha—in each of the ACI stages in nonresponders.

**Table 4:** Nonresponder Differentially Abundant Proteins at the Time of Stage 2 vs Stage 1^*a*^

Protein With Differential Abundance		iTRAQ Proteomics		Label-free Proteomics	
Description	Accession	Fold Change^*b*^	*P* Value^*c*^	Fold Change^*b*^	*P* Value^*c*^
**iTRAQ proteomics**					
Hemoglobin subunit beta	P68871	−12.8	**5.72E-06**	−2.6	.222
Coagulation factor XIII B chain	P05160	−7.0	**2.79E-12**	−1.3	.580
C-reactive protein	P02741	−6.0	**.026**	−2.0	.154
Complement C3	P01024	−4.4	**1.78E-15**	1.2	.400
Vitamin D-binding protein	P02774	−4.3	**.001**	1.1	.942
Coagulation factor XIII A chain	P00488	−4.1	**4.79E-05**	−1.2	.516
Haptoglobin	P00738	−3.9	**.001**	−1.3	.154
Histidine-rich glycoprotein	P04196	−3.4	**.001**	1.1	.974
Alpha-2-macroglobulin	P01023	−3.0	**6.14E-08**	−1.2	.665
Complement factor H-related protein 5	Q9BXR6	−2.8	**.005**	1.0	.982
Alpha-1-antitrypsin	P01009	−2.4	**.018**	−1.1	.429
Serotransferrin	P02787	−2.2	**7.79E-09**	1.1	.605
Thrombospondin-1	P07996	−2.2	**5.14E-08**	−1.8	.356
Phosphatidylinositol-glycan-specific phospholipase D	P80108	−2.1	**.005**	−1.2	.358
Properdin	P27918	−2.1	**.037**	1.3	.646
Multimerin 1	Q13201	−2.0	**.050**	−1.4	.726
Complement factor H	P08603	2.1	**.001**	1.0	.715
Cartilage acidic protein 1	Q9NQ79	2.2	**.017**	1.2	.612
Heparin cofactor 2	P05546	2.4	**.006**	1.1	.596
Complement C1r subcomponent	P00736	2.5	**.008**	−1.1	.432
Coagulation factor V	P12259	2.5	**.001**	−1.1	.628
Complement C1q subcomponent subunit A	P02745	2.6	**.021**	1.2	.381
Proteoglycan 4	Q92954	2.6	**.017**	−1.1	.731
Gelsolin	P06396	3.0	**.019**	1.0	.664
Beta-Ala-His dipeptidase	Q96KN2	3.0	**.001**	1.3	.205
Inter-alpha-trypsin inhibitor heavy chain H2	P19823	3.1	**2.64E-05**	1.2	.467
Complement C1q subcomponent subunit B	P02746	3.2	**.011**	−1.0	.827
Vitamin K–dependent protein S	P07225	3.2	**.001**	−1.0	.684
Alpha-2-HS-glycoprotein	P02765	3.5	**.008**	1.0	.809
Phosphatidylcholine-sterol acyltransferase	P04180	3.5	**.026**	1.1	.475
Coagulation factor XII	P00748	3.7	**.002**	−1.0	.785
Cartilage oligomeric matrix protein	P49747	3.8	**.001**	−1.3	.378
Inter-alpha-trypsin inhibitor heavy chain H4	Q14624	4.1	**.001**	1.1	.159
Ig gamma-1 chain C region	P01857	4.2	**.028**	1.0	.776
Lysozyme C	P61626	4.4	**.004**	−1.9	.291
Apolipoprotein C-I	P02654	4.5	**.012**	−1.2	.600
Complement C1s subcomponent	P09871	4.8	**4.35E-08**	−1.0	.830
Prothrombin	P00734	4.8	**5.95E-06**	−1.0	.774
Clusterin	P10909	5.1	**.000**	−1.1	.624
Ceruloplasmin	P00450	5.3	**8.20E-08**	1.1	.561
Apolipoprotein L1	O14781	6.2	**.013**	−1.2	.600
Pigment epithelium-derived factor	P36955	6.5	**.006**	−1.7	.691
Pregnancy zone protein	P20742	6.9	**.008**	2.3	.741
Apolipoprotein E	P02649	7.9	**5.98E-08**	−1.0	.673
Apolipoprotein A-II	P02652	8.7	**7.05E-05**	1.0	.807
Endoplasmin	P14625	8.8	**.005**	1.0	.133
Apolipoprotein C-II	P02655	12.4	**.002**	−1.0	.745
Apolipoprotein A-IV	P06727	14.7	**7.78E-12**	−1.0	.579
**Label-free proteomics**					
Immunoglobulin kappa variable 1-5	P01602	1.0	**.980**	−24.1	**.048**
Hemoglobin subunit alpha	P69905	Not identified	—	−3.6	**.049**

a Proteins that were identified by iTRAQ or label-free LC-MS/MS as differentially abundant (≥±2.0-fold change; *P* < .05; protein identified by at least 2 unique peptides) in the plasma of clinical nonresponders at stage 2 vs stage 1. Proteins identified with iTRAQ LC-MS/MS (pooled samples) were subsequently assessed in the label-free LC-MS/MS data set of matched nonpooled samples. iTRAQ, isobaric tagging for relative and absolute quantitation; LC-MS/MS, liquid chromatography–tandem mass spectrometry.

b Positive numbers denote higher abundance at stage 1 vs stage 2.

c Bold *P* values denote significant findings.

Many of the proteins identified in the iTRAQ proteomics that differed between surgical procedures in nonresponders were proteins involved in the acute-phase response: alpha-1 antitrypsin, apolipoprotein AII, apolipoprotein E, ceruloplasmin, clusterin, complement factor H, gelsolin, heparin cofactor 2, and inter-alpha heavy chains (H1, H2, and H4) (Table 4).

### Measurement of Candidate Protein Concentrations Using ELISA

**Figure 2.**
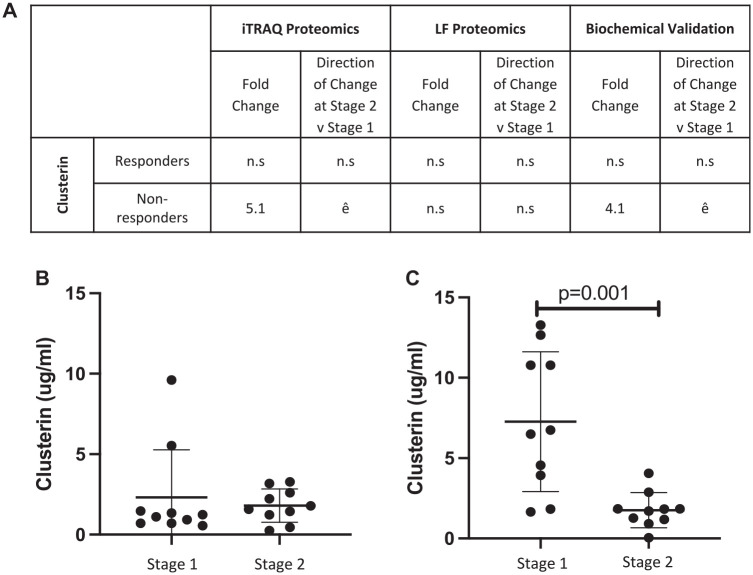
Biochemical validation of a differentially abundant protein (clusterin) identified using isobaric tagging for relative and absolute quantitation (iTRAQ) proteomics. (A) The differential abundance of human clusterin as measured by iTRAQ mass spectrometry and biochemical ELISA. Quantitative ELISA confirmed that clusterin (B) is not differentially abundant before chondrocyte implantation (stage 2) as compared with cartilage harvest surgery (stage 1) in responders but (C) is increased in the plasma at stage 1 vs stage 2 in ACI nonresponders (*P* = .001; Student *t* test). Mean ± SD are presented. ACI, autologous chondrocyte implantation; ELISA, enzyme-linked immunosorbent assay; iTRAQ, isobaric tagging for relative and absolute quantitation; NR, nonresponders; R, responders.

**Figure 3.**
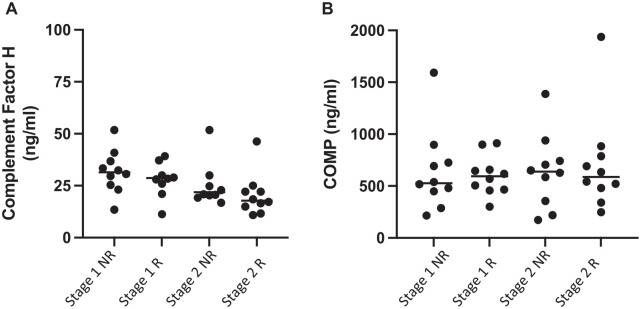
Attempted biochemical validation of 2 differentially abundant proteins—(A) complement factor H and (B) COMP—identified using iTRAQ proteomics. (A) No differential abundance of complement factor H as measured by iTRAQ mass spectrometry and biochemical ELISA. Quantitative ELISA also confirmed that (B) COMP is not differentially abundant in plasma collected at stages 1 and 2 or in relation to clinical response to ACI. Horizontal line indicates median. ACI, autologous chondrocyte implantation; COMP, cartilage oligomeric matrix protein; ELISA, enzyme-linked immunosorbent assay; iTRAQ, isobaric tagging for relative and absolute quantitation; NR, nonresponders; R, responders.

The iTRAQ LC-MS/MS result of decreased plasma clusterin concentration at stage 2 versus stage 1 surgery in nonresponders could be confirmed biochemically using ELISA (stage 1, 7.27 ± 4.35 μg/mL; stage 2, 1.76 ± 1.09 μg/mL; *P* = .001; Student *t* test) (Figure 2). Biochemical assessment confirmed that this differential protein abundance between the surgical procedures could not be detected in the plasma of individuals who responded to ACI (stage 1, 2.32 ± 2.94 μg/mL; stage 2, 1.81 ± 1.03 pg/mL; *P* = .481; Student *t* test). However, the other proteins selected for immunoassay that were identified from the iTRAQ LC-MS/MS analysis, COMP and complement factor H, did not replicate the iTRAQ data (Figure 3).

## Discussion

This study is the first to attempt to determine if there is a difference in the plasma proteome of individuals who either respond or demonstrate clinical failure after treatment of osteochondral/chondral defects with ACI. The initial approach was to assess pooled samples using a cheaper, quicker proteomic technique: iTRAQ LC-MS/MS. The results from this initial proteomic investigation were promising, particularly as we had anticipated difficulty in being able to detect protein differences within the systemic circulation that reflected joint environment changes indicative of an individual's capacity for cartilage repair. After the iTRAQ analysis, we were encouraged that the greatest number of protein differences were identified in response to cartilage harvest surgery (ie, at stage 2 vs stage 1) in the ACI nonresponders, as this replicated our previous findings in the synovial fluid of these same patients.^11,12^ Moreover, several of these proteins related to acute-phase response signaling, a key biological pathway that we have associated with the synovial fluid of nonresponders, who have a poor clinical outcome to the treatment.^11^ To examine this finding in more depth, we undertook an independent analysis of plasma samples (some matched to the iTRAQ analysis) with LF LC-MS/MS to analyze each sample individually. In performing this nonpooled analysis, we demonstrated that there were very few proteins differentially abundant, either between clinical response groups preoperatively or between the stages of surgery, irrespective of clinical outcome.

Regardless of the limited number of proteins that were differentially abundant between clinical response groups in the LF LC-MS/MS data, 1 protein (cartilage acidic protein 1) was identified as having potential to differentiate individuals, before treatment, who are likely to respond well to ACI or not. Identification of a biomarker that can be measured in the plasma preoperatively to predict clinical outcome remains the ultimate goal in biomarker discovery for prediction of cartilage repair surgery.^14^ The benefits of being able to measure a marker within the plasma include the ease of sample acquisition and the fact that these samples could be obtained in the outpatient or primary care setting, informing clinical decision-making regarding who should be treated with expensive ACI.

Cartilage acidic protein 1 is a glycosylated extracellular matrix protein that is expressed in the deep zone of articular cartilage.^29^ Xianpeng et al^34^ demonstrated that human articular chondrocytes and synovial fibroblasts upregulate cartilage acidic protein 1 expression in response to stimulation by proinflammatory cytokines. Moreover, in mice, there was a female-specific association of cartilage acidic protein 1 with OA progression. Interestingly, cartilage acidic protein 1 was recently identified in a study of nearly 40,000 individuals as an OA-associated plasma protein with the capacity to predict the likelihood of progression to joint replacement.^31^ Our data add weight to this finding, suggesting that this marker can differentiate those whose OA is likely to progress regardless of therapeutic intervention, at least with cell therapy. In future work, cartilage acidic protein 1 should be assessed in a larger cohort of cell therapy patients, including a wider array of clinical failures and successes (not only extremes of response) to assess the potential of this protein to differentiate individuals whose outcomes may be more difficult to predict on the basis of other injury and demographic parameters. Furthermore, assessing this marker in patients being treated with other routine orthopaedic surgical procedures aimed at preventing OA progression would help to determine its prospect in the presurgery screening of patients who are destined to fail or progress to joint replacement regardless of the intervention. Moreover, it should be noted that the clinical success or failure of these patients was assessed with the Lysholm score, which is a patient-reported outcome measure that assesses aspects of pain and function.^19,27^ The Lysholm score has been used to assess ACI efficacy at our specialist center for many years; however, it would be interesting to determine whether cartilage acidic protein 1 can differentiate responders and nonresponders based on an appropriate minimal clinically important difference for other, more widely used outcome measures (eg, Knee injury and Osteoarthritis Outcome Score) in an independent cohort.

The data presented in this study highlight the importance of validating proteins in nonpooled samples where pooled proteomic analyses are used. Despite there being differences in the numbers of differentially abundant proteins that were detected and quantitated across the independent proteomic data sets, the iTRAQ data were limited by identified proteins that could not be validated using ELISA. Neither COMP nor complement factor H replicated the iTRAQ findings when assessed via biochemical assay, perhaps instead providing support for the nonpooled LF LC-MS/MS data in which no differences were observed for these analytes.

Proteomic profiling of plasma has long been recognized as technically challenging with several options for pretreating plasma samples such that lower-abundance protein differences can be detected, each with benefits and limitations.^8^ To overcome the complex mix of plasma proteins, we attempted to use ProteoMiner beads to compress the dynamic range of proteins with hexapeptide technology.^5^ Despite this approach being used before both proteomic analyses, the method of eluting the peptides from the beads differed. For the samples utilized for iTRAQ analyses, these proteins were eluted after dynamic range compression using the kit reagents, whereas for LF LC-MS/MS, an on-bead trypsin digest was performed. These different techniques were used as the proteins that were eluted from the beads for the iTRAQ analysis then had to be pooled before steps to prepare the samples for MS, whereas analysis of individual samples meant that the trypsin digests could be performed on the ProteoMiner beads. It may be that the efficiency of sample removal from the beads differed between these approaches or, perhaps more likely, the pooling of equal amounts of ProteoMiner bead–treated proteins before trypsin digest may contribute to the differential proteomic results in this study. Furthermore, slightly different patient samples were used for the 2 analyses to ensure that non–freeze/thawed samples could be analyzed. The inclusion of slightly different patients may have also contributed to our not being able to identify as many differentially abundant proteins in the nonpooled group, as it may be that there were outliers that contributed to the changes being identified in the pooled iTRAQ analysis.

Biochemical analysis, however, did confirm the iTRAQ LC-MS/MS findings that in nonresponders to ACI, clusterin was reduced at the time of stage 2 surgery versus presurgery. Clusterin is a heterodimeric glycoprotein that is implicated in inhibition of the complement cascade.^25^ A recent review article explored clusterin as a biomarker for early OA,^16^ particularly as the plasma and synovial fluid concentrations of this protein are associated with radiographic severity of OA^32^ and it is hypothesized to be a marker of synovial inflammation.^2^ This may indicate that patients who do not respond to ACI may have more advanced OA or perhaps they have a more inflammatory response to cartilage harvest surgery (stage 1), which contributes to their poor clinical response.

Although we have identified some plasma proteins that have been linked with OA progression and severity, notably many of the widely studied biomarkers, such as CD163 and soluble hyaluronan,^3,17^ were not identified in the proteomic analyses. Therefore, there may be value in using targeted biomarker identification of biologically relevant proteins that are undetectable with the described LC-MS/MS techniques, complementary to the unbiased proteomic approaches, to screen for candidate predictive markers in the plasma.

In conclusion, this study provides evidence that differential abundance of proteins within the plasma exists and has the potential to differentiate between ACI clinical outcome groups at baseline and during treatment. Cartilage acid protein 1 is the most promising baseline differentiator identified in this study, with potential utility in presurgery screening and personalized medicine. However, a follow-up study is required to validate this finding in a larger ACI group and ideally in an independent setting, utilizing appropriate statistical analyses to determine whether this marker can add predictive value alongside all of the known baseline predictors of ACI, such as age, sex, preoperative function, concomitant procedures, and defect characteristics. Furthermore, there is potential that any biomarkers identified in this study may have the capacity to predict outcome to other therapies to treat early OA.
